# Association between lid margin collarettes and dry eye disease severity in the Dry Eye Assessment and Management (DREAM) study

**DOI:** 10.1038/s41433-025-04105-5

**Published:** 2025-11-25

**Authors:** Alexander E. Azar, Eliot N. Haddad, Patrick A. Augello, Meng C. Lin, Gui-Shuang Ying, Penny A. Asbell, Rony R. Sayegh, Dry Eye Assessment and Management (DREAM) Study Research Group

**Affiliations:** 1 Case Western Reserve University School of Medicine, Cleveland, OH USA; 2 Cleveland Clinic Lerner College of Medicine, Cleveland, OH USA; 3 Department of Ophthalmology, Perelman School of Medicine, University of Pennsylvania, Philadelphia, PA USA; 4 College of Public Health, Temple University, Philadelphia, PA USA; 5 Herbert Wertheim School of Optometry & Vision Science, University of California, Berkeley, CA USA; 6 University of Memphis, Memphis, TN USA; 7 Cole Eye Institute, Cleveland Clinic, Cleveland, OH USA

**Keywords:** Corneal diseases, Eyelid diseases, Eye manifestations, Predictive markers

## Abstract

**Background/objectives:**

Lid margin collarettes are a distinct clinical finding and their association with meibomian gland dysfunction (MGD) and dry eye symptoms and signs remains uncertain. We leverage the large cohort of subjects in the Dry Eye Assessment and Management (DREAM) study data to elucidate collarettes’ impact on dry eye disease (DED).

**Subjects/methods:**

We performed secondary analysis of DREAM study data, a multicentre, double-blinded clinical trial evaluating omega-3 supplementation in patients with moderate-to-severe DED. DED symptoms (Ocular Surface Disease Index [OSDI] and Ocular Discomfort) and signs (conjunctival staining, corneal staining, tear break-up time (TBUT), Schirmer test, MGD grade) were compared by collarette presence and severity. Data from all visits (baseline, months 3, 6, 12) were assessed and adjusted for demographics and baseline comorbidities.

**Results:**

Of 1070 eyes at baseline, 65.3% had no collarettes, 28.0% had 1–5, and 6.6% had 6–20. Over the 1 year period of the study, 67% of eyes had no change in collarette severity, while 19% had increased severity and 14% had decreased severity. Collarettes were more common in Whites (82.1% vs 69.9%, *p* = 0.001), non-Hispanics (92.3% vs 83.2%, *p* = 0.006), and those with facial rosacea (25.0% vs 17.7%, *p* = 0.05). Multivariate analysis showed collarettes were associated with increased corneal staining (5.06 vs 4.59, *p* = 0.01), decreased TBUT (3.24 vs 3.54 s, *p* = 0.01), decreased Schirmer test (7.92 vs 8.63, *p* = 0.04), more severe eyelid erythema (18.6% vs 12.2%, *p* < 0.001), and higher composite DED sign severity (0.51 vs 0.49, *p* = 0.03). However, they were not associated with conjunctival staining, MGD grade, or DED symptoms (*p* ≥ 0.30). Similar findings were observed in comparisons of collarette severity. Subjects with collarettes had lower tear levels of IL-1β (*p* = 0.04), IL-10 (*p* = 0.02), and INF-γ (*p* = 0.001).

**Conclusions:**

Collarettes were common in patients with moderate-to-severe DED, and most do not progress over time. Although they were associated with worse ocular surface signs, notably eyelid erythema, no association was found with dry eye symptoms. Our results will help physicians counsel and guide the approach to treating dry eye patients with collarettes, and generate pilot data for tear film biomarker identification.

## Introduction

Lid margin collarettes refer to debris found at the base of the eyelashes. They are associated with chronic blepharitis [1]. Collarettes are classified as cylindrical sleeves versus scales. The former have been reported to be pathognomonic for *Demodex* blepharitis, with some studies showing that between 50% and 90% of patients with cylindrical dandruff will have *Demodex* [2–5]. Collarettes can also be formed from *Staphylococcus*, and they are found more distally on the lash and have a scaly, golden-yellow appearance [1, 2]. *Propionibacterium acnes* and *Pityrosporum ovale* have also grown from eyelid cultures and likely contribute to colarette formation [6]. Though these organisms have a homeostatic role, overgrowth and infestation can lead to increased inflammation [1, 5, 7].

Chronic blepharitis is associated with increased microbial proliferation and collarette production, leading to inflammation, lid erythema, foreign body sensation, and itching [2, 3, 7, 8]. As the disease progresses further, other signs may develop, including conjunctival injection, lid margin oedema, lash loss or irregularity, meibomian gland dysfunction (MGD), or corneal staining [2, 7, 8]. A knowledge gap persists in our understanding of the prevalence and role of collarettes, including the correlation between their presence and dry eye symptoms. In fact, it is common for patients to have collarettes without having any clinical symptoms of blepharitis [2, 7].

This study investigates the prevalence of collarettes in patients with dry eye disease and evaluates whether the presence of lid margin collarettes is associated with the severity of dry eye signs and symptoms in patients with moderate-to-severe dry eye who participated in the Dry Eye Assessment and Management Study (DREAM).

## Materials and methods

We performed a secondary analysis of data from the DREAM study, a multicentre, double-blinded clinical trial on patients with moderate-to-severe dry eye disease designed to evaluate the efficacy and safety of omega-3 fatty acid supplementation in treating DED [9, 10]. In the DREAM study, patients were included based on the presence of both signs (e.g. conjunctival staining, corneal fluoresceine staining, tear film break-up time) and symptoms, including the Ocular Surface Disease Index (OSDI), with dry eye-related symptoms lasting at least 6 months. A total of 535 patients were 2:1 randomised to an active omega-3 supplement group or an olive oil placebo and then followed over a 12 month period.

During the DREAM study visits, clinicians were asked to evaluate lid margin debris on both upper and lower eyelids of each eye, and grade it as normal (0 collarettes), mild (1–5 collarettes), moderate (6–20 collarettes, a few fragments), severe (21–40 collarettes, 1–2 clumps), and very severe (40+ collarettes; more than 3 clumps). The measures for DED signs evaluated in each eye in the DREAM study included eyelid erythema, conjunctival lissamine green staining, corneal fluorescein staining, TBUT, Schirmer test, MGD grading of plugging and secretions, tear osmolarity, and a composite DED severity score of signs. Eyelid erythema was evaluated by the clinician as normal, mild, moderate, or severe based on redness and neovascularisation of the upper and lower eyelid margins. Corneal staining and conjunctival staining were used to assess ocular surface damage, with 5 areas of the cornea being graded 0 to 3 (maximum score of 15 per eye), and 2 areas of the conjunctiva being graded 0 to 3 (maximum score of 6 per eye). The TBUT with fluorescein assessed tear film stability by measuring the time (in seconds) required for the appearance of tear film breakup during an inter-blink period, with a shorter TBUT suggesting a more unstable tear film. TBUT was measured three times and averaged. The Schirmer test with anaesthetics was used to assess tear volume by placing a paper strip in the lower eyelid for 5 min and then measuring the wetting length, with a shorter length indicating reduced tear volume. MGD grading was the total score of Meibomian gland plugging and lid secretion, which was graded on a 0 to 3 scale. Tear osmolarity was measured in the inferior tear lake with a TearLab Osmolarity System (*n* = 405), measuring the concentration of solutes (mOsml/L) in the tear film, with a higher score being more abnormal. The composite signs severity score consisted of five dry eye signs, namely TBUT, Schirmer, corneal and conjunctival staining scores, and MGD grade. Each of the five signs was transformed to a common unit severity score from 0 to 1, where 0 indicates no DED and 1 indicates the most severe DED, according to the discrete severity grading system of the DEWS report. Scores between the quartile points were linearly interpolated. A composite sign severity score was calculated per eye by taking the mean of the severity scores of the five independent signs.

Dry eye symptoms were evaluated using the OSDI. Question 3 of the Brief Ocular Discomfort Inventory (BODI) asking the patient to rate their average ocular discomfort over the past week on a 0 to 10 scale was also included for analysis. The OSDI is a 12-item questionnaire that assesses a patient’s ocular surface symptoms, with three categories of questions: vision-related function, ocular symptoms, and environmental triggers. Patients rated how often they experienced each symptom on a scale from 0 (they do not experience it) to 4 (they experience it all the time).

The detailed methodology of tear collection and cytokine measurement in the DREAM study has been previously described [11, 12]. Of the 535 participants in the study, 244 were from sites without –80 °C freezers for storage of tear samples and thus could not participate in tear cytokine analysis. Of the remaining 291 participants at sites with 80 °C freezers, only 131 tear samples were collected that were eligible for statistical analysis of tear cytokines (pooled tear volume of ≥4 µL) [11, 12]. Tear samples were collected via microcapillary tubes from each eye and then pooled for each visit date, at baseline, month 6, and month 12. Pooling ensured sufficient volume for valid analysis and was not expected to affect the overall cytokine profile [12, 13]. Cytokine concentrations were measured using the MILLIPLEX-MAG kit (High Sensitivity Human Cytokine Kit, Cat # HSCYTMAG-28SK, Millipore Corporation, Billerica, MA 01821). For immune cell analysis, samples were collected from 1049 eyes of 527 subjects. Conjunctival impression cytology was done on these samples to identify the proportion of HLA-DR-positive cells within total cells, epithelial cells, and white blood cells. The detailed methodology of sample collection and impression cytology has been published previously [14].

### Statistical methods

Categorical measures were presented as frequencies with percentages and compared using the chi-squared test or Fisher's exact test, as appropriate. Continuous measures (i.e. age) were presented with a mean and standard deviation (SD) if the data were normally distributed, and using median (1st quartile, 3rd quartile) for the skewed data. Analyses of patient baseline characteristics and dry eye symptoms were performed by comparing subject-level characteristics between subjects with collarettes in either eye vs no collarettes in both eyes, using a two-sample *t* test for comparing means or Wilcoxon Rank Sum test for comparing medians. Comparisons of dry eye signs between eyes with vs without collarettes were performed at eye level, where collarettes from each eye of a patient at each visit were included into the analysis and inter-eye correlation and correlation from repeated measures were accounted for by using generalised estimating equations. Longitudinal prevalence of collarettes was calculated. Univariate and multivariate analyses for comparisons of dry eye symptoms and signs were performed at the 0, 3, 6, and 12-month time points. All multivariate analyses were adjusted by age, gender, race, smoking status, visit, and comorbidities (Sjögren syndrome, facial rosacea, rheumatoid arthritis, peripheral artery disease, depression) that were found to be associated with dry eye symptoms and signs in the DREAM study [15]. Tear cytokines were measured from pooled tears from two eyes of a participant; thus, comparisons of cytokines were performed at the subject level between participants with versus without collarettes using the Wilcoxon rank sum test and the Kruskal-Wallis test due to skewed cytokine data. Immune cells were measured at eye level; thus, comparisons of immune cells were performed at eye level by using clustered Wilcoxon rank sum test to account for inter-eye correlation. Statistical analyses were conducted using R, version 4.2 (RStudio, Boston MA), and SAS version 9.4 (SAS Institute Inc., Cary, NC). Two-sided *p* < 0.05 was considered to be statistically significant.

## Results

This study involved 535 participants from 27 clinical sites, with a mean age of 58 years (SD = 13.2 years). The majority were female (434 participants, 81%) and White (398 participants, 74%). Follow-up was completed by 479 participants (90%) at the 6-month visit and 486 participants (91%) at the 12-month visit.

At baseline, 699 (65%) eyes had no collarettes, 300 (28%) had mild, and 71 (7%) had moderate collarettes, and no eyes had severe or very severe collarettes at baseline (Table 1). The frequency distribution of collarettes was stable at 6 and 12-month follow-up visits, with the emergence of a small number of eyes with severe and very severe collarettes (Table 1). Of the 976 eyes that were examined from baseline to the 12-month visit, 67% of eyes had no change in collarette severity, while 19% had increased severity and 14% had decreased severity. The agreement in the presence and severity of collarettes between the two eyes was high, with 339 (63%) patients without collarettes in either eye, 175 (33%) had collarettes in both eyes, 8 (1%) had collarettes in the right eye only, and 13 (2%) in the left eye only. The percent agreement between the two eyes in collarettes was 94.95% at baseline, 94.22% at 3 months, 93.55% at 6 months, and 94.25% at 12 months. Weighted Kappa (95% CI) was 0.90 (0.86, 0.94) at baseline, 0.88 (0.84, 0.92) at 3 months, 0.85 (0.80, 0.90) at 6 months, and 0.88 (0.84, 0.92) at 12 months.

**Table 1:** Frequency of collarettes over time.

Collarettes	Baseline (*N* = 1070 eyes)	3 months (*N* = 1004 eyes)	6 months (*N* = 962 eyes)	12 months (*N* = 976 eyes)
Normal (0)	699 (65.33%)	657 (65.44%)	669 (69.54%)	686 (70.29%)
Mild (1–5)	300 (28.04%)	292 (29.08%)	227 (23.60%)	224 (22.95%)
Moderate (6–20)	71 (6.64%)	48 (4.78%)	58 (6.03%)	57 (5.84%)
Severe (21–40)	0	7 (0.70%)	8 (0.83%)	7 (0.72%)
Very Severe (40+)	0	0	0	2 (0.20%)

Associations between patient characteristics and collarettes at baseline are shown in Table 2. Presence of collarettes was significantly associated with White race (*p* = 0.001), non-Hispanic ethnicity (*p *= 0.006), and rosacea (*p* = 0.05, Table 2). Baseline collarettes were not significantly associated with age (*p* = 0.06), gender (*p* = 0.11), smoking status (*p* = 0.45), dry eye treatment, and self-reported medical history (all *p* > 0.05).

**Table 2:** Comparison of baseline characteristics between participants with or without collarettes in any eye at baseline.

Baseline Characteristic (*n* = 535 participants)	With Collarettes (*n* = 183 participants)	Without Collarettes (*n* = 352 participants)	*P* value
Age, median (Q1, Q3)	60.0 (54.0, 68.0)	59.0 (51.0, 66.0)	0.06
Sex^a^			0.09
*Female*	151 (77.0%)	283 (83.5%)	
*Male*	45 (23.0%)	56 (16.5%)	
Race			**0.001**
*Black or African American*	21 (10.7%)	43 (12.7%)	
*White*	161 (82.1%)	237 (69.9%)	
*Other*^*b*^	14 (7.1%)	59 (17.4%)	
Ethnicity			**0.006**
*Hispanic*	14 (7.1%)	54 (15.9%)	
*Non-Hispanic*	181 (92.3%)	282 (83.2%)	
*Unable to answer*	1 (0.5%)	3 (0.9%)	
Cigarette Smoking			0.86
Never	135 (68.9%)	232 (68.4%)	
Former	53 (27.0%)	89 (26.3%)	
Current	8 (4.1%)	18 (5.3%)	
Self-Reported Medical History			
Sjögren’s syndrome	19 (9.7%)	37 (10.9%)	0.77
Thyroid dysfunction	45 (23.0%)	63 (18.6%)	0.26
Hypertension	55 (28.1%)	100 (29.5%)	0.77
Diabetes	22 (11.2%)	40 (11.8%)	0.89
Rheumatoid arthritis	13 (6.6%)	36 (10.6%)	0.16
Irritable bowel	20 (10.2%)	39 (11.5%)	0.67
Osteoarthritis	60 (30.6%)	81 (23.9%)	0.10
Hypercholesterolemia	74 (37.8%)	117 (34.5%)	0.46
Depression	43 (21.9%)	79 (23.3%)	0.75
Rosacea	49 (25.0%)	60 (17.7%)	**0.05**
Peripheral artery disease	23 (11.7%)	31 (9.1%)	0.37
Dry Eye Disease Treatment			
Artificial tears or gel	156 (79.6%)	271 (79.9%)	0.91
Cyclosporine drops	79 (40.3%)	127 (37.5%)	0.52
Warm lid soaks	41 (20.9%)	72 (21.2%)	1.00
Lid scrubs	18 (9.2%)	44 (13.0%)	0.21
Baby shampoo	12 (6.1%)	21 (6.2%)	1.00

Bold values indicate statistical significance *p* < 0.05.

*Q*1 first quartile, *Q*3 third quartile.

^a^Sex and race were self-reported by participant.

^b^Other includes Asian, American Indian, Alaskan Native, more than once race, or unable to answer.

In the analysis with all visit time points combined, collarettes were not significantly associated with dry eye symptoms measured by OSDI total score (*p* = 0.81), subscale scores (all *p* > 0.56), and BODI ocular discomfort score (*p* = 0.30, Table 3). Similarly, the severity level of collarettes was not significantly associated with dry eye symptoms (all *p* > 0.15).

**Table 3:** Comparison of dry eye symptoms between participants with and without collarettes, with various collarette severity, across all time points.

Outcome	Collarettes	Baseline (*N* = 1070 eyes)	3 months (*N* = 1004 eyes)	6 months (*N *= 962 eyes)	12 months (*N* = 976 eyes)	All time combined (*N* = 4012 eyes)
OSDI score, mean (SD)	No (0)	42.10 (1.87)	30.90 (3.30)	32.59 (2.42)	32.13 (2.89)	34.45 (2.11)
	Yes (>0)	42.85 (1.99)	30.43 (3.35)	33.05 (2.77)	28.41 (3.29)	34.20 (2.16)
	*P* value^a^	0.61	0.78	0.79	**0.03**	0.81
	Normal (0)	41.55 (1.90)	30.87 (3.32)	32.52 (2.43)	32.13 (2.89)	34.32 (2.12)
	Mild (1–5)	43.89 (2.07)	30.50 (3.38)	33.38 (2.82)	28.66 (3.38)	34.70 (2.19)
	Moderate or above (6+)	35.29 (2.72)	29.86 (4.25)	31.56 (3.73)	27.57 (3.80)	31.43 (2.74)
	*P* value^a^	**0.003**	0.93	0.81	0.08	0.24
	Linear trend *P* value^a^	0.50	0.74	0.91	**0.02**	0.38
Vision-related function	No (0)	28.96 (2.43)	25.72 (2.97)	24.76 (3.16)	24.90 (3.47)	26.04 (2.12)
	Yes (>0)	29.51 (2.63)	24.02 (3.11)	25.88 (3.53)	21.15 (3.88)	25.56 (2.23)
	*P* value	0.76	0.35	0.57	**0.05**	0.68
	Normal (0)	28.53 (2.52)	25.72 (2.98)	24.73 (3.17)	24.90 (3.47)	25.97 (2.13)
	Mild (1–5)	30.31 (2.70)	24.03 (3.13)	26.04 (3.56)	21.02 (4.02)	25.85 (2.24)
	Moderate or above (6+)	23.65 (3.95)	23.98 (4.33)	25.11 (4.71)	21.60 (4.21)	23.97 (3.00)
	*P* value	0.13	0.65	0.83	0.14	0.65
	Linear trend *P* value	0.67	0.35	0.72	**0.03**	0.39
Ocular symptoms	No (0)	53.45 (2.49)	35.14 (3.80)	40.87 (2.73)	39.09 (2.71)	42.19 (2.33)
	Yes (>0)	53.51 (2.58)	34.57 (3.86)	40.69 (3.13)	34.89 (3.22)	41.49 (2.39)
	*P* value	0.97	0.77	0.93	**0.04**	0.57
	Normal (0)	52.57 (2.57)	35.10 (3.82)	40.78 (2.74)	39.08 (2.70)	41.96 (2.35)
	Mild (1–5)	55.14 (2.74)	34.66 (3.88)	41.11 (3.16)	35.97 (3.34)	42.37 (2.43)
	Moderate or above (6+)	41.59 (3.44)	33.90 (5.16)	38.74 (4.20)	31.22 (3.96)	36.60 (3.10)
	*P* value	**<0.001**	0.94	0.77	**0.03**	0.06
	Linear Trend *P* value	0.13	0.82	0.74	**0.01**	0.15
Environmental triggers	No (0)	56.28 (3.90)	38.23 (5.36)	39.15 (6.29)	40.84 (4.27)	43.71 (3.54)
	Yes (>0)	59.07 (4.06)	40.69 (5.44)	38.85 (6.74)	37.41 (4.68)	44.68 (3.64)
	*P* value	0.24	0.34	0.91	0.22	0.56
	Normal (0)	55.91 (3.87)	38.24 (5.37)	39.04 (6.29)	40.83 (4.28)	43.64 (3.55)
	Mild (1–5)	59.74 (4.05)	40.66 (5.44)	39.43 (6.82)	36.92 (4.82)	44.97 (3.64)
	Moderate or above (6+)	53.97 (5.80)	40.91 (7.45)	36.19 (7.98)	39.05 (6.28)	43.05 (4.88)
	*P* value	0.25	0.63	0.83	0.43	0.69
	Linear trend *P* value	0.52	0.39	0.94	0.48	0.71
Ocular discomfort (BODI question 3)^b^	No (0)	48.11 (2.43)	36.02 (3.46)	38.65 (2.86)	40.54 (2.92)	39.91 (2.26)
	Yes (>0)	47.79 (2.59)	35.02 (3.51)	39.95 (3.35)	36.75 (3.46)	38.70 (2.36)
	*P* value	0.85	0.60	0.51	0.07	0.30
	Normal (0)	47.50 (2.48)	35.80 (3.50)	38.52 (2.85)	40.53 (2.91)	40.60 (2.25)
	Mild (1–5)	48.93 (2.68)	35.53 (3.57)	40.54 (3.45)	37.53 (3.51)	40.99 (2.42)
	Moderate or above (6+)	39.49 (3.89)	30.87 (5.24)	37.24 (4.06)	34.10 (4.74)	35.53 (3.38)
	*P* value	**0.03**	0.49	0.51	0.15	0.13
	Linear Trend *P* value	0.23	0.42	0.73	0.12	0.26

Bold values indicate statistical significance *p* < 0.05.

*OSDI* Ocular Surface Discomfort Index, *BODI* Brief Ocular Discomfort Inventory.

^a^Adjusted by age, gender, race, smoking status, visit, comorbidities (Sjögren syndrome, facial rosacea, rheumatoid arthritis, peripheral artery disease, depression).

^b^BODI#3: on a scale from 0 to 10, please rate your ocular discomfort by circling the one number that best describes your ocular discomfort on the average in the last week.

In multivariate analysis, DED signs were significantly more severe in eyes with collarettes, with a higher composite DED severity score in the collarette group (0.51 vs 0.49, *p* = 0.03, Table 4). Of the dry eye signs analysed, presence of collarettes was associated with worse scores for eye lid erythema (18.6% vs 12.2% with moderate or severe eyelid erythema, *p* < 0.001), corneal staining (5.06 vs 4.59, *p* = 0.01), TBUT (3.24 vs 3.54 s, *p* = 0.01), and Schirmer test score (7.92 vs 8.63, *p* = 0.04). The presence of collarettes was not significantly associated with conjunctival staining score (*p* = 0.41) and MGD grade (*p* = 0.12). Collarettes were associated with an unexpectedly better tear osmolarity (304.3 vs 307.0, *p* = 0.01).

**Table 4:** Comparison of dry eye signs between eyes with and without collarettes across all time points.

Dry eye sign (*n* = 4012 eyes)	With collarettes (*n* = 1301 eyes)	Without collarettes (*n* = 2711 eyes)	*P* value^1^
Eyelid erythema			**<0.001**
*No erythema*	451 (34.67%)	1384 (51.05%)	
*Mild*	608 (46.73%)	996 (36.74%)	
*Moderate*	236 (18.14%)	321 (11.84%)	
*Severe*	6 (0.46%)	10 (0.37%)	
Conjunctival staining	2.82 (0.51)	2.89 (0.50)	0.41
Corneal staining	5.06 (1.07)	4.59 (1.06)	**0.01**
Tear break-up time (TBUT) (s)	3.24 (0.44)	3.54 (0.44)	**0.01**
Schirmer test (mm)	7.92 (0.86)	8.63 (0.82)	**0.04**
Meibomian gland dysfunction (MGD)	4.12 (0.26)	3.95 (0.25)	0.12
Tear osmolarity^2^(mOsmol/L)	304.27 (6.48)	307.04 (6.44)	**0.01**
Composite dry eye disease severity score based on signs^3^	0.51 (0.06)	0.49 (0.06)	**0.03**

Bold values indicate statistical significance *p* < 0.05.

^1^Adjusted by age, gender, race, smoking status, visit, and comorbidities (Sjögren’s syndrome, facial rosacea, rheumatoid arthritis, peripheral artery disease, depression).

^1^Generalised estimating equation was used to account for inter-eye correlation.

^2^Tear osmolarity not recorded during the month 3 visit.

^3^Composite severity score does not include eyelid erythema or tear osmolarity.

Though DED signs were significantly more severe in eyes with collarettes, not all signs were linearly associated with greater collarette severity. Scores for eyelid erythema (*p* < 0.001), TBUT (*p* < 0.001), MGD grade (*p* = 0.01), and overall composite DED severity score (*p* = 0.01) became more severe as the severity of collarettes increased (Supplementary Table 1). Conjunctival staining, corneal staining, and Schirmer test were not significantly associated with collarette severity (all *p* > 0.05). Tear osmolarity was unexpectedly better as collarette severity increased.

In a small subset of subjects with tear cytokines measures available at baseline, 6 months or 12 months (*n* = 327 patient visits), subjects with collarettes had lower levels of IL-1β (*p* = 0.04), IL-10 (*p* = 0.02), and INF-γ (*p* = 0.001, Table 5). Other cytokines did not significantly vary between the groups. There were no significant associations between collarettes and immune cells collected by impression cytology (all *p* > 0.45, Supplementary Table 2).

**Table 5:** Comparison of cytokines between participants with and without collarettes across all time points.

Cytokines	Median (1st quartile, 3rd quartile)		*P*-value^1^
	With collarettes	Without collarettes	
IL-1β (pg/mL)	1.13 (0.00, 4.00)	2.86 (0.00, 4.55)	**0.04**
IL-6 (pg/mL)	4.63 (3.02, 8.64)	5.03 (3.00, 8.77)	0.48
IL-8 (pg/mL)	37.04 (14.48, 83.23)	34.23 (12.33, 97.10)	0.54
IL-17A (pg/mL)	5.14 (1.86, 10.28)	7.92 (2.64, 12.28)	0.053
IL-10 (pg/mL)	5.05 (0.00, 16.19)	9.03 (0.00, 18.20)	**0.02**
INF-γ (pg/mL)	14.62 (6.80, 27.31)	24.10 (9.76, 40.96)	**0.001**
TNF-α (pg/mL)	0.00 (0.00, 2.19)	0.00 (0.00, 3.12)	0.43

Bold values indicate statistical significance *p* < 0.05.

*IL* Interleukin, *INF* Interferon, *TNF* Tumour Necrosis Factor.

^1^*P* values obtained from nonparametric tests.

## Discussion

We present findings from a large-scale study (*n* = 535) analysing collarettes in patients with moderate-to-severe DED with standardised data collection. This approach allows for a robust evaluation of associations between lid margin collarettes and various DED markers. Our study found that among patients with moderate to severe DED, those with collarettes were more likely to be White and non-Hispanic. The racial difference was most pronounced when comparing the White versus Other groups, though the Black/African American group also had a significantly lower percentage of collarettes compared to the White group. It is unclear whether this represents a true racial difference or whether collarettes are more visible over lighter skin.

We found a significant association between collarettes and rosacea. This association has been reported in the ophthalmologic and dermatologic literature. Microbial overgrowth has been implicated in various dermatological diseases, one of those being facial and ocular rosacea [7, 16, 17]. The mechanism by which they are associated is not fully understood, but the current consensus is that there are changes to the skin flora that allow for microbial overgrowth [3, 7, 17]. Patients with rosacea have been shown to have altered skin flora, revealing increased density of *Demodex*, *Staphylococcus epidermis*, and other non-flora bacteria [16]. However, it is not clear whether this altered flora is caused by the rosacea or independent risk factors that lead to changes in the flora environment, leading to the development of rosacea.

Another possible explanation for the increased prevalence of collarettes in White, non-Hispanic patients is that rosacea is more common in individuals with lighter skin tones, especially Fitzpatrick 1 and 2 skin types [16]. Furthermore, skin phototype and sunlight exposure are factors that can change the flora environment, thus allowing for overgrowth of *Demodex* and *Staphylococcus* organisms [7]. Given this association, it is possible that those with lighter skin are at higher risk for rosacea and flora changes, thus they are more likely to present with collarettes. However, other reports suggest that rosacea is equally prevalent in pigmented individuals, but often misdiagnosed or underdiagnosed, given the difficulty of discerning erythema and telangiectasia in dark skin [18]. As such, additional research is needed to determine the underlying cause of the association between race and collarettes, including the role of skin phototype, genetics, and other potential confounders.

Regardless of the mechanism by which facial rosacea and altered skin flora develop, it is hypothesised that microbial overgrowth on the face leads to migration and proliferation around the eyes, and overgrowth on lashes promotes ocular rosacea and/or blepharitis [7, 17]. The mites and the bacteria that they harbour then trigger inflammatory/immune reactions, causing the ocular rosacea and blepharitis [7, 17]. This pathophysiologic mechanism is supported by our findings as the collarettes were associated with more severe eyelid erythema, a common symptom of chronic blepharitis. Additionally, our findings also support a recent report that lower eyelid margin redness, along with other clinical parameters, can predict blepharitis diagnosis with a prediction accuracy of 74% [19].

Another demographic factor commonly associated with collarettes is age, but no significant correlation was found between collarettes and age in this study. Generally, the prevalence and density of *Demodex* infestation have been shown to increase with age [3, 7]. This increase typically occurs in patients between 50 and 70 years old and is thought to occur due to age-related weakening of the immune system and an increase in skin fragility, thus allowing the mite to penetrate more rapidly [3]. Due to these factors, we expected to find a significant association between the presence of collarettes and age. The mean age of the collarette group was about 2.6 years older than the non-collarette group (59.4 vs 57.3, *p* = 0.07, Table 2), but the association was not strong enough to claim a significant relationship. Significance may not have been observed in our study due to the utilisation of the DREAM patient database. DREAM patients were all selected based on a diagnosis of moderate to severe dry eye disease, and this sample may be biased towards older patients, as they generally have more severe disease.

Among participants with moderate to severe DED, those with collarettes generally had increased severity of DED signs but did not have any changes in ocular symptoms. Analysis of overall OSDI scores, OSDI subset scores, and BODI Question 3 (ocular discomfort level) showed no significant differences in patients with and without collarettes. However, DED signs were significantly more severe in patients with collarettes when looking at the composite dry eye severity score and many of the individual signs, including eyelid erythema, corneal staining, TBUT, and Schirmer test. While statistically significant, the difference in eyelid erythema between the two groups is the sign that is most clinically significant. Interestingly, there was no significant difference in MGD grading when comparing collarettes vs no collarettes, but MGD grade linearly increased as the severity of collarettes increased (*p* = 0.01, Supplementary Table 1). This association with moderate-to-severe collarettes may indicate that MGD is more pronounced in more severe cases and is generally unchanged in mild vs normal cases. This distinction is important for clinicians deciding whether to pursue MGD-targeted therapies based on collarette findings.

There are few studies looking at the impact of collarettes on DED severity, and these were focused on *Demodex* infestations. When patients with MGD-related DED in China were observed, those with *D. folliculorum* infestation were found to have increased scores of corneal staining, lid margin abnormalities, and MGD, alongside a shorter TBUT [20]. Another study of newly diagnosed DED patients in Turkey found increased Schirmer test scores in subjects with *Demodex* infestation [21]. Higher amounts of *Demodex* organisms have also been associated with more severe signs, such as meibomian gland loss and keratitis [22]. Furthermore, reduction of collarettes with lotilaner ophthalmic solution was also shown to lead to lower levels of erythema [23].

There is more literature regarding the isolated effects of collarettes, which lead to inflammation, eyelid erythema, lash loss and irregularity, functional tear deficiency, and corneal staining [2, 8, 24]. When analysing tear film cytokines, we expected to see increased inflammatory markers in the collarettes group due to the inflammatory nature of blepharitis. Interleukin-10 (IL-10), an anti-inflammatory cytokine, was decreased in the collarette group, and thus represents increased susceptibility to inflammation (*p* = 0.02, Table 5). Our results also showed an unexpected decrease in the pro-inflammatory markers, IL-1β and INF-γ, which have been shown in the literature to be elevated in DED [25]. One possible explanation is that a deficiency in IFN-γ may predispose to *Demodex* or *Staphylococcal* infestation, as IFN-γ is known to have anti-microbial effects and be protective against parasite infection [26].

Though the presence of collarettes correlated with more severe DED signs, these signs did not noticeably affect patient symptoms in our study. When comparing groups with and without collarettes, there were no differences in symptoms as measured by the OSDI and BODI. Similarly, comparison by severity of collarettes did not show any difference in DED symptoms in patients with no, mild, or moderate-to-severe collarettes. Therefore, both the presence of and severity of collarettes do not have a significant effect on DED symptom severity, despite worsened signs. This lack of correlation highlights the difference between anterior and posterior blepharitis, the latter being more associated with evaporative dry eye symptoms. This raises the question of which patient groups should be treated for their collarettes. While our results suggest that treating the collarettes in patients in the US with moderate-to-severe DED would result in improvement of some signs but not symptoms, one study found that both ocular signs and symptoms in cases of blepharitis improved with a reduction in collarettes after treatment with ivermectin 1.0% ointment [24, 27]. It is also conceivable that in this cohort of moderate to severe dry eye patients, a multitude of underlying causes for ocular discomfort may obscure the relationship between the presence or severity of collarettes and the BODI. In addition, the limited breadth of symptoms included in the OSDI includes grittiness, soreness/pain, blurred/poor vision, and sensitivity to light. Symptoms such as burning, stinging, itching, and redness, typically associated with blepharitis, are not included in the OSDI questionnaire. As such, this study may highlight the inadequacy of the OSDI to capture the symptoms associated with the presence of collarettes, and that different questionnaire tools have different sensitivities for various aspects of DED.

In summary, data from the DREAM study provided a unique opportunity to evaluate lid margin collarettes. The DREAM study was a randomised, double-blinded, multi-centre clinical trial of 535 well-characterised patients with moderate-to-severe DED and is one of the few large cohort studies to evaluate collarettes. Drawing on this data allowed us to conduct an analysis of lid margin collarettes in DED patients and reveal multiple associations. While dry eye symptoms remained largely unchanged between those with and without collarettes, certain DED signs were more severe in the presence of collarettes. We also identified potential risk factors associated with collarettes and showed that tear cytokine levels may be altered in patients with collarettes. These findings warrant further investigation to understand the mechanisms by which collarettes contribute to dry eye disease. One must keep in mind the limitations of our study, which did not describe the collarette type, and only focused on the DED symptoms that are included in the OSDI, and the average level of ocular discomfort over the past week. As such, more research is needed to elucidate the effect of collarettes and their treatment on dry eye symptoms.

## Summary

### What is known about this topic

- Lid margin collarettes are a hallmark of chronic blepharitis and have been linked to microbial overgrowth (e.g. Demodex, Staphylococcus), but their clinical significance in dry eye disease (DED) remained unclear.
- Previous small studies suggested a potential link between collarettes and ocular surface inflammation, but few evaluated these relationships in large, well-defined DED cohorts.

### What this study adds

- In this DREAM study analysis (*n* = 535), collarettes were linked to worse DED signs, including higher corneal staining (5.06 vs. 4.59, *p* = 0.01), lower TBUT (3.24 vs. 3.54 s, *p* = 0.01), and increased eyelid erythema (18.6% vs. 12.2%, *p* < 0.001).
- Collarettes were not associated with symptom severity (OSDI: 34.20 vs. 34.45, *p* = 0.81; BODI: 38.70 vs. 39.91, *p* = 0.30), indicating a signs–symptoms mismatch. Patients with collarettes had lower tear levels of IL-1β (*p* = 0.04), IL-10 (*p* = 0.02), and IFN-γ (*p* = 0.001), which may have implications for the pathophysiology of collarettes in DED.

## Supplementary information

Supplementary Table 1

Supplementary Table 2

Credit Roster for the Dry Eye Assessment And Management (DREAM) Study

## Data Availability

De-identified data from the Dry Eye Assessment and Management (DREAM) study are available through the National Eye Institute (NEI) Data Commons (https://neidatacommons.nei.nih.gov/) to researchers who submit an approved data use proposal.
