# Supplementary material for: Association between lid margin collarettes and dry eye disease severity in the Dry Eye Assessment and Management (DREAM) study
Source: Eye (Lond). 2025 Nov 25;40(1):139–46. doi: 10.1038/s41433-025-04105-5 (PMC12764526; doi:10.1038/s41433-025-04105-5)
Supplement: Supplementary file 1 — Supplementary Table 1 [file 41433_2025_4105_MOESM1_ESM.docx]

**Supplementary Table 1**: Comparison of dry eye signs based on collarette severity across all time points

| **Dry eye signs (n=4012 eyes)** | **Normal (0 collarettes)**  **(n=2711 eyes)** | **Mild (1-5 collarettes)**  **(n=1043 eyes)** | **Moderate or above (6+ collarettes)**  **(n=258 eyes)** | **Linear trend p value^1^** |
| --- | --- | --- | --- | --- |
| **Eyelid erythema^2^** |  |  |  | **<0.001** |
| *No erythema* | 1384 (51.05%) | 397 (38.06%) | 54 (20.93%) |  |
| *Mild* | 996 (36.74%) | 499 (47.84%) | 109 (42.25%) |  |
| *Moderate* | 321 (11.84%) | 145 (13.90%) | 91 (35.27%) |  |
| *Severe* | 10 (0.37%) | 2 (0.19%) | 4 (1.55%) |  |
| **Conjunctival staining** | 2.88 (0.50) | 2.86 (0.51) | 2.59 (0.52) | 0.14 |
| **Corneal staining** | 4.57 (1.05) | 5.15 (1.07) | 4.53 (1.11) | 0.10 |
| **Tear break-up time (TBUT)** (sec) | 3.52 (0.44) | 3.33 (0.44) | 2.75 (0.47) | **<0.001** |
| **Schirmer test** (mm) | 8.65 (0.81) | 7.85 (0.86) | 8.28 (1.03) | 0.07 |
| **Meibomian gland dysfunction (MGD)** | 3.98 (0.25) | 4.01 (0.27) | 4.72 (0.33) | **0.01** |
| **Tear osmolarity^3^**  (mOsmol/L) | 306.87 (6.42) | 304.89 (6.47) | 301.56 (6.53) | **<0.001** |
| **Composite dry eye disease severity score based on signs^4^** | 0.49 (0.06) | 0.51 (0.06) | 0.52 (0.06) | **0.01** |

^1^ Adjusted by age, gender, race, smoking status, visit, comorbidities (Sjögren syndrome, facial rosacea, rheumatoid arthritis, peripheral artery disease, depression).

^2^ Generalised estimating equations (GEE) used to account for inter-eye correlation for eye lid erythema

^3^ Tear osmolarity not recorded during month 3 visit.

^4^ Composite severity score does not include eyelid erythema or tear osmolarity
