# Supplementary material for: Association between lid margin collarettes and dry eye disease severity in the Dry Eye Assessment and Management (DREAM) study
Source: Eye (Lond). 2025 Nov 25;40(1):139–46. doi: 10.1038/s41433-025-04105-5 (PMC12764526; doi:10.1038/s41433-025-04105-5)
Supplement: Supplementary file 2 — Supplementary Table 2 [file 41433_2025_4105_MOESM2_ESM.docx]

**Supplementary Table 2:** Comparison of immune cells between eyes with and without collarettes across all time points

|  | **Median (1^st^ quartile, 3^rd^ quartile)** | |  |
| --- | --- | --- | --- |
| **Immune cells** | **With collarettes** | **Without collarettes** | **P-value^1^** |
| **Number of gated cells** | 15079.00 (8468.00, 23351.00) | 14801.00 (8684.00, 23129.00) | 0.83 |
| **Percentage of dendritic cells in white blood cells (WBC)** | 66.70 (42.60, 86.50) | 69.90 (44.40, 87.20) | 0.71 |
| **Percentage of T-cells in WBC** | 37.04 (14.48, 83.23) | 34.23 (12.33, 97.10) | 0.54 |
| **Percentage of T-toxic cells in WBC** | 8.30 (3.30, 15.10) | 8.30 (2.90, 15.60) | 0.96 |
| **Percentage of Treg cells in WBC** | 0.00 (0.00, 0.60) | 0.00 (0.00, 0.30) | 0.66 |
| **Percentage of Th1 cells in WBC** | 0.00 (0.00, 0.00) | 0.00 (0.00, 0.00) | 0.62 |
| **Percentage of Th17 cells in WBC** | 0.00 (0.00, 0.00) | 0.00 (0.00, 0.00) | 0.45 |

^1^P values obtained from Wilcoxon Rank Sum while accounting for inter-eye correlation.
